# Intensive care unit-acquired urinary tract infections in a regional critical care system

**DOI:** 10.1186/cc3023

**Published:** 2005-01-06

**Authors:** Kevin B Laupland, Sean M Bagshaw, Daniel B Gregson, Andrew W Kirkpatrick, Terry Ross, Deirdre L Church

**Affiliations:** 1 Assistant Professor, Departments of Critical Care Medicine, Pathology and Laboratory Medicine, and Community Health Services, Center for Anti-microbial Resistance, Calgary Health Region, Calgary Laboratory Services, and the University of Calgary, Calgary, Alberta, Canada; 2 Fellow, Departments of Medicine and Community Health Services, Calgary Health Region, Calgary Laboratory Services, and the University of Calgary, Calgary, Alberta, Canada; 3 Associate Professor, Departments of Pathology and Laboratory Medicine, and Medicine, Calgary Health Region, Calgary Laboratory Services, and the University of Calgary, Calgary, Alberta, Canada; 4 Clinical Assistant Professor, Departments of Critical Care Medicine, and Surgery, Calgary Health Region and the University of Calgary, Calgary, Alberta, Canada; 5 Analyst, Center for Anti-microbial Resistance, Calgary Health Region, Calgary Laboratory Services, and the University of Calgary, Calgary, Alberta, Canada; 6 Professor, Departments of pathology and Laboratory Medicine, and Medicine, Calgary Health Region, Calgary Laboratory services, and the University of Calgary, Calgary, Alberta, Canada

**Keywords:** incidence, intensive care unit, mortality, urinary tract infection

## Abstract

**Introduction:**

Few studies have evaluated urinary tract infections (UTIs) specifically acquired within intensive care units (ICUs), and the effect of such infections on patient outcome is unclear. The objectives of this study were to describe the occurrence, microbiology, and risk factors for acquiring UTIs in the ICU and to determine whether these infections independently increase mortality.

**Methods:**

A surveillance cohort study was conducted among all adults admitted to multi-system and cardiovascular surgery ICUs in the Calgary Health Region (CHR, population about 1 million) between 1 January 2000 and 31 December 2002.

**Results:**

During the 3 years, 4465 patients were admitted 4915 times to a CHR ICU for 48 hours or more. A total of 356 ICU-acquired UTIs (defined as at least 10^5 ^colony-forming units/ml of one or two organisms 48 hours or more after ICU admission) occurred among 290 (6.5%) patients, yielding an overall incidence density of ICU-acquired UTIs of 9.6 per 1000 ICU days. Four bacteremic/fungemic ICU-acquired UTIs occurred (0.1 per 1000 ICU days). Development of an ICU-acquired UTI was more common in women (relative risk [RR] 1.58; 95% confidence interval [CI] 1.43–1.75; *P *< 0.0001) and in medical (9%) compared with non-cardiac surgical (6%), and cardiac surgical patients (2%). The most common organisms isolated were *Escherichia coli *(23%), *Candida albicans *(20%), and *Enterococcus *species (15%). Antibiotic-resistant organisms were identified among 14% isolates. Although development of an ICU-acquired UTI was associated with significantly higher crude in-hospital mortality (86/290 [30%] vs. 862/4167 [21%]; RR = 1.43; 95% CI 1.19–1.73; *P *< 0.001); an ICU-acquired UTI was not an independent predictor for death.

**Conclusions:**

Development of an ICU-acquired UTI is common in critically ill patients. Although a marker of increased morbidity associated with critical illness, it is not a significant attributable cause of mortality.

## Introduction

Infection of the urinary tract (UTI) is the most common hospital-acquired infection in North America and is among the most frequent nosocomial infections in critically ill patients [1-4]. Nosocomial UTIs have been associated with a threefold increased risk for mortality in hospital-based studies, with estimates of more than 50,000 excess deaths occurring per year in the USA as a result of these infections [5]. Furthermore, in several studies nosocomial UTIs have been associated with increased length of hospital stay and cost [6,7]. Despite their importance, there have only been very limited studies focused on nosocomial UTIs in the critically ill. Richards and colleagues reported on intensive care unit (ICU) nosocomial infections in the National Nosocomial Infections Surveillance System (NNIS) database and found that UTI was responsible for 20–30% of nosocomial infections in medical/surgical ICUs [1,8]. Finklestein and colleagues determined an incidence of 10–14 UTI per 1000 catheter days among 337 patients in a single Israeli ICU [9]. Rosser and colleagues retrospectively reviewed 126 trauma ICU patients with sepsis and found that increased length of stay, length of catheterization, and age (more than 60 years) were independent factors associated with the development of nosocomial UTI [10]. These studies were limited in part either as a result of being conducted in specialized ICUs or critically ill patient subsets, by small sample size, or by limited assessment of outcome.

We previously conducted a study of all patients admitted to multidisciplinary ICUs in the Calgary Health Region (CHR) during a 1 year period and found that increased length of stay and female gender were independently associated with the acquisition of these infections [2]. However, this study was limited by exclusion of many cardiovascular surgery patients and like other investigations had insufficient statistical power to detect a clinically important increased risk for mortality associated with ICU-acquired UTIs [11]. We therefore conducted further surveillance among all critically ill adults in the CHR to better delineate the occurrence, microbiology, and risk factors for acquiring ICU-acquired UTIs and determine whether these infections increase the risk for mortality.

## Methods

### Study population

The CHR administers all acute hospital care to the residents of the cities of Calgary and Airdrie and several large adjacent regions (population about 1 million). All ICUs within the CHR are closed units staffed by fully trained intensivists and are administered by the Department of Critical Care Medicine, University of Calgary, and CHR. These include a 14-bed cardiovascular surgery ICU (CVICU) and multidisciplinary ICUs (total 44 beds) at each of the three adult acute care centers in the CHR. All patients 18 years of age and older admitted to an adult multidisciplinary ICU or the CVICU in the CHR for at least 48 hours during 1 January 2000 and 31 December 2002 were included in the study. The Conjoint Health Research Ethics Review Board at the University of Calgary approved the study.

### Protocol

The study used a cohort design that linked data from regional administrative, critical care, and microbiology databases. Demographic, clinical, basic laboratory, and outcome data were obtained from all patients admitted to ICUs in the CHR using the ICU Tracer database [12]. Calgary Laboratory Services, a region-based laboratory that handles all routine bacterial specimens from CHR patients, identified all relevant positive culture results. Data from the source databases were linked on the basis of unique hospital numbers using Access 2002 (Microsoft Corp., Redmond, WA).

### Definitions

An ICU-acquired UTI was defined using a modification of the criteria of Costel and colleagues as those patients with a positive urine culture (at least 100,000 colony-forming units/ml of one or two organisms) first identified on ICU day 3 (48 hours) or later [2,13]. Patients with positive urine cultures within 48 hours of ICU discharge were also considered to have ICU-acquired UTIs. A bacteremic/fungemic UTI was defined as a UTI with a concomitantly positive blood culture with the same organism within a 48 hour period [2]. A surgical patient was any patient recorded as having an operative diagnosis or admitted from the trauma ward, post cardiac surgery care unit, or directly from the operating room. Severity of illness and intensity of care at admission were assessed using the Acute Physiology and Chronic Health Evaluation II (APACHE II) and the Therapeutic Intervention Scoring System (TISS) scores, respectively [14,15]. Shock was deemed to be present if a vasopressor infusion was required. Laboratory testing was performed in accordance with standard guidelines as described previously [2] with the exception that, as of June 2001, only cultures either positive by screening by an ATPase–luciferase assay or by specific physician request were cultured [16]. Antimicrobial-resistant organisms were defined as methicillin-resistant *Staphylococcus aureus*, vancomycin-resistant *Enterococcus faecalis *or *faecium*, or any Gram-negative organisms resistant to one or more of ciprofloxacin, tobramycin/gentamicin, ceftazidime, piperacillin, or imipenem.

### Statistical analysis

Analysis was performed with Stata version 8.0 (Stata Corp, College Station, TX). The occurrence of ICU-acquired UTI was expressed as (1) the cumulative incidence of patients with at least one UTI episode per admittance to ICU, and (2) the incidence density based on the number of ICU-acquired UTI episodes per total patient days of ICU stay. Normally or near-normally distributed variables were reported as means and standard deviations, and non-normally distributed variables as medians with inter-quartile ranges (IQRs). Means were compared with Student's *t*-test and medians with the Mann–Whitney *U*-test. Differences in proportions between categorical data were assessed with the χ^2 ^or Fisher's exact test. A multivariable logistic regression model was developed to identify independent risk factors for mortality associated with these infections, with the use of backward stepwise variable elimination. Variables included those identified in our previous study [2] and those found to be significant to the *P *≤ 0.1 level in univariate analysis. Final model discrimination was assessed by using the area under the receiver operator characteristic curve and calibration by using the Hosmer–Lemeshow goodness-of-fit test. *P *< 0.05 was considered significant for all comparisons unless otherwise stated.

## Results

### Demographics

During the 3 years of the study, 4465 patients were admitted 4915 times to a CHR ICU for 48 hours or more. Twenty-five percent (1099) of admissions were to the CVICU. Sixty-one percent (2709) of the patients were male, the mean age was 61.2 ± 17.4 years, and the mean APACHE II scores were 26.1 ± 8.3 points. In all, 1975 (45%) were classified as medical patients.

### Incidence of ICU-acquired UTI

A total of 356 ICU-acquired UTIs occurred among 290 (7%) patients during surveillance. Three hundred and three ICU-acquired UTIs were on first ICU admission episodes (that is, 13 patients fulfilled criteria for a second UTI during their first ICU stay) and 43 were on second, 9 on third, and 1 on fifth ICU admission episodes. The overall incidence density of ICU-acquired UTI was 9.6 per 1000 ICU days. Only four ICU-acquired UTIs were associated with a positive blood culture with the same organism for an overall incidence density of bacteremic/fungemic ICU-acquired UTI of 0.1 per 1000 ICU days. The overall incidence of ICU-acquired UTI was significantly (*P *≤ 0.01) higher in the year 2000 (143/1531; 9%) than in 2001 (112/1651; 7%) or 2002 (101/1733; 6%).

### Factors associated with the development of an ICU-acquired UTI

Several factors present at admission to ICU were associated with increased incidence of ICU-acquired UTI. Women (174/1755), in comparison with men (116/2709), were at significantly increased risk (relative risk [RR] = 1.58; 95% confidence interval [CI] 1.43–1.75; *P *< 0.0001) for development of an ICU-acquired UTI. A significantly different rate of development of these infections was observed among admission categories, with an incidence of 9% (181/1975) in medical patients, 6% (89/1391) in non-cardiovascular surgical patients, and 2% (20/1099) in cardiovascular surgical patients (*P *< 0.001 overall; and *P *< 0.005 for each pairwise comparison). No differences were observed between patients who developed an ICU-acquired UTI with regard to either mean age or APACHE II score, although patients who developed an ICU-acquired UTI had lower mean admission Therapeutic Intervention Scoring System scores than those patients who did not develop one of these infections (41.6 ± 15.1 versus 45.1 ± 17.3 points; *P *< 0.01).

A significant association between ICU length of stay and development of an ICU-acquired UTI was observed. The median length of ICU stay among patients with ICU-acquired UTI was 12.0 (IQR 5.7–21.0) days compared with 4.1 (IQR 2.8–7.5) days for those without (*P *< 0.0001). Similarly an increased overall median hospital length of stay was associated with development of an ICU-acquired UTI (30 days, IQR 16–62; 16 days, IQR 9–29; *P *< 0.0001).

### Microbiology

The median time from ICU admission to development of a first UTI was 7.0 (IQR 4.2–12.1) days. Most (337/356; 95%) of the UTIs were monomicrobial infections but in 19 cases two organisms were identified simultaneously at 10^8 ^colony-forming units/l or more. The organisms causing ICU-acquired UTI are shown in Table 1. Antibiotic-resistant organisms were identified in 14% (53/375) of isolates. These organisms were *Escherichia coli *in 29 (55%), *Pseudomonas aeruginosa *in 12 (23%), *Klebsiella *species in 5 (9%), and other Gram-negative enterics in 7 (13%); none were vancomycin-resistant *Enterococcus faecalis *or *faecium *or methicillin-resistant *Staphylococcus aureus*. Among these antibiotic-resistant organisms, resistance occurred to ciprofloxacin in 33% (17/52), gentamicin in 21% (11/52), tobramycin in 9% (5/53), ceftazidime in 13% (7/53), piperacillin in 61% (31/51), and piperacillin/tazobactam in 12% (6/51). Imipenem resistance was identified in one of seven isolates of *Pseudomonas aeruginosa *tested (susceptibility testing for carbapenems only started routinely in 2002). Resistance to two different classes of antimicrobials occurred in 13 isolates, and 3 isolates were resistant to three different classes.

### Mortality

Development of an ICU-acquired UTI was associated with a significantly higher crude ICU-related mortality (52/290 [18%] versus 519/4175 [12%]; RR = 1.44; 95% CI 1.11–1.89; *P *= 0.01) and overall in-hospital mortality (86/290 [30%] vs. 862/4167 [21%]; RR = 1.43; 95% CI 1.19–1.73; *P *< 0.001)] than those who did not develop this infection. A multivariable logistic regression model (*n *= 4434) that had both good discrimination (area under receiver operator characteristic curve = 0.75) and calibration (goodness-of-fit *P *= 0.08) was developed to assess risk factors for in-hospital death. After controlling for other significant covariates, ICU-acquired UTI was not independently associated with death, as shown in Table 2.

## Discussion

We observed an incidence density of ICU-acquired UTI of 9.6 per 1000 ICU days that is comparable to that observed in other studies that evaluated nosocomial UTIs in ICUs [2,9,10,17]. However, an important strength of this study is that all patients admitted to adult ICUs (both academic-based and community-based) in a large region were included. As a result, this study should be representative of many critically ill populations at large and the results more widely generalizable. Previous studies have been limited to single specialized medical, surgical, or combined medical–surgical ICUs [9,10] or in series of selected ICUs participating in surveillance systems [1,8]. Our previous study, which included all multidisciplinary ICUs, was limited in part because we failed to include many cardiovascular surgical patients [2]. As demonstrated by our observation of a significant difference in risk of acquiring ICU-acquired UTI between cardiac surgical, non-cardiac surgical, and medical patients, care must be paid to patient 'case-mix' in comparing between studies of these infections. It is noteworthy that we did not exclude non-residents of the CHR in this study despite the fact that we have previously argued for such a practice [18]. Given that we did not observe any significant rate differences among CHR residents and non-residents (data not shown), that the population at risk was restricted to those admitted to CHR ICU (and not the entire base population of the CHR), and that the mortality outcome for those with a ICU-acquired UTI was not related to residency status, we pooled our entire patient cohort for analysis.

There are several possible explanations for our important observation of a lower rate of ICU-acquired UTIs in the latter 2 years of the study. The first possibility is that heightened awareness from our first report [2] or concomitant preventive efforts (such as a large regional quality improvement initiative to reduce ventilator-associated pneumonia) with increased attention to the use of medical devices and attention to hand washing among staff could have had a role. Anecdotally, we feel it is unlikely to be related to a decreased use of urinary catheters because we estimate that nearly all (more than 90%) of our patients ill enough to require ICU admission for 2 or more days have an indwelling urinary catheter. A second possibility for the reduced rate in the latter years of the study is that there might have been increased use of systemic antimicrobials active against urinary pathogens. This is only speculative because we do not have actual data to support this possibility. A third consideration is that physicians less frequently ordered urine cultures in the second and third years of the study such that the overall culture positivity rate was less. Unlike in our first study, in which we collected data on negative cultures [2], we did not have access to such results in the present study and are therefore unable to assess this. The fourth possibility, and the one that we suspect might be the most important reason for the reduced rate of ICU-acquired UTIs being diagnosed in the latter part of the study, is that we changed our laboratory testing practice in June 2001. At that point a bacteriuria screening assay was implemented regionally after demonstrating its utility in outpatients [16]. Since that time only urine samples either positive by that assay or by specific physician request are cultured. On the basis of the sensitivity of the assay of 86%, it is expected that a 10–15% reduction in the rate of culture positivity would occur with its implementation. However, a more important influence is that this assay does not detect yeast because it is based on the specific detection of bacterial ATP [16,19]. *Candida *species are among the most important causes of ICU-acquired UTI and a reduced rate of ICU-acquired UTI is expected if these organisms are not routinely cultured. We are currently planning a study to evaluate the optimal laboratory means of identifying ICU-acquired UTIs in our region.

The most clinically important and novel finding of this study was that ICU-acquired UTIs do not independently increase the risk for death among patients admitted to ICUs. Unlike in all previous studies potentially able to investigate this question, the present study was adequately powered to detect a clinically significant increased mortality risk [2,9,10]. Although we did observe that these infections increased the crude mortality risk, once confounding for measures of severity of disease, diagnostic category, and length of ICU stay were controlled for, ICU-acquired UTI was not significantly associated with death. This contrasts with the findings of Platt and colleagues, which showed that nosocomial UTIs were associated with a significant attributable mortality in a general hospital population [5]. It is clinically important to ascertain whether ICU-acquired UTIs are associated with attributable mortality because there may be implications for treatment. Although practice variation among different intensivists and between ICUs in our region probably exists, we commonly withhold antimicrobial therapy for bacteriuria or funguria in the absence of an associated clinical infective syndrome. Although a randomized, prospective, clinical trial is required to address optimal practice, our current observations of a low rate of bacteremic/fungemic ICU-acquired UTI and lack of attributable mortality suggests that a clinical judgment-based approach to treatment may be reasonable.

## Conclusion

We present the results of a large observational cohort study that confirms that critical illness is commonly complicated by the development of a nosocomial UTI. Although these infections are crudely associated with death they are not associated with a significantly increased attributable mortality. Further studies are needed for better definition of the potential adverse effect of these infections on patient morbidity and cost to the healthcare system.

## Key messages

• 	A surveillance cohort study was conducted among all adults admitted to multi-system and cardiovascular surgery ICUs in the Calgary Health Region (population about 1 million) during a 3 year period.

• 	ICU-acquired UTI commonly (7%) complicated the course of patients admitted to ICU for greater than 48 hours; women and medical patients were at highest risk.

• 	Development of an ICU-acquired UTI was not an independent risk factor for in-hospital mortality.

## Abbreviations

APACHE = Acute Physiology and Chronic Health Evaluation; CHR = Calgary Health Region; CI = confidence interval; CVICU = cardiovascular surgery intensive care unit; ICU = intensive care unit; IQR = inter-quartile range; RR = relative risk; UTI = urinary tract infection.

## Competing interests

The author(s) declare that they have no competing interests.

## Authors' contributions

KBL conceived and designed the study, conducted the principal analysis, and drafted the manuscript. SMB participated in the study design and revision of the manuscript. DBG, TR, and DLC contributed to data collection and manuscript revision. AWK assisted with analysis and manuscript revision. All authors read and approved the final manuscript.

## Figures and Tables

**Table 1:** Microbial etiologies of ICU-acquired urinary tract infections, Calgary, Alberta, Canada

Organism	Occurrence (%)^a^
*Escherichia coli*	87 (23)
*Candida albicans*	74 (20)
*Enterococcus *species	57 (15)
*Pseudomonas aeruginosa*	34 (10)
*Candida glabrata*	20 (5)
*Klebsiella *species	20 (5)
Coagulase-negative staphylococci	18 (5)
*Proteus mirabilis*	17 (5)
*Candida *other^b^	16 (4)
*Enterobacter *species	10 (3)
*Citrobacter *species	5 (1)
*Staphylococcus *aureus	4 (1)
*Streptococcus *species	3 (1)
Other^c^	10 (3)

^a^The number of isolates (*n *= 375) exceeds the number of patients with ICU-acquired UTI because some patients had more than one UTI episode or had polymicrobial infection.

^b^*Candida krusei *(1), *C. lusitaniae *(1), *C. tropicalis *(4), *Candida *not speciated (10).

^c^Includes *Lactobacillus *species (1), *Proteus penneri *(1), *Serratia marcescens *(2), *Morganella morganii *(2), *Providencia rettgeri *(1), *Hafnia alvei *(2) and *Staphylococcus saphrophyticus *(1).

ICU, intensive care unit; UTI, urinary tract infection.

**Table 2:** Multivariable logistic modeling of variables associated with in-hospital death among patients admitted for at least 48 hours to a Calgary Health Region intensive care unit, 2000–2002

Variable	Odds ratio (95% CI)	*P*
Admission APACHE II score (per point)	1.09 (1.08–1.10)	<0.001
Logarithm of ICU length of stay (per day)	1.31 (1.18–1.45)	<0.001
Non-cardiac surgical patient^a^	0.78 (0.66–0.92)	<0.01
Cardiac surgical patient^a^	0.14 (0.11–0.18)	<0.001
ICU-acquired urinary tract infection	1.02 (0.76–1.37)	0.9

^a^Compared with a reference group of medical patients.

APACHE II, Acute Physiology and Chronic Health Evaluation II; CI, confidence interval; ICU, intensive care unit.

## References

[B1] Richards MJ, Edwards JR, Culver DH, Gaynes RP (2000). Nosocomial infections in combined medical-surgical intensive care units in the United States. Infect Control Hosp Epidemiol.

[B2] Laupland KB, Zygun DA, Davies HD, Church DL, Louie TJ, Doig CJ (2002). Incidence and risk factors for acquiring nosocomial urinary tract infection in the critically ill. J Crit Care.

[B3] Haley RW, Culver DH, White JW, Morgan WM, Emori TG (1985). The nationwide nosocomial infection rate. A new need for vital statistics. Am J Epidemiol.

[B4] Erbay H, Yalcin AN, Serin S, Turgut H, Tomatir E, Cetin B, Zencir M (2003). Nosocomial infections in intensive care unit in a Turkish university hospital: a 2-year survey. Intensive Care Med.

[B5] Platt R, Polk BF, Murdock B, Rosner B (1982). Mortality associated with nosocomial urinary-tract infection. N Engl J Med.

[B6] Centers for Disease Control (1992). Public health focus: Surveillance, prevention, and control of nosocomial infections. MMWR.

[B7] Givens CD, Wenzel RP (1980). Catheter-associated urinary tract infections in surgical patients: a controlled study on the excess morbidity and costs. J Urol.

[B8] Richards MJ, Edwards JR, Culver DH, Gaynes RP (1999). Nosocomial infections in medical intensive care units in the United States. National Nosocomial Infections Surveillance System. Crit Care Med.

[B9] Finkelstein R, Rabino G, Kassis I, Mahamid I (2000). Device-associated, device-day infection rates in an Israeli adult general intensive care unit. J Hosp Infect.

[B10] Rosser CJ, Bare RL, Meredith JW (1999). Urinary tract infections in the critically ill patient with a urinary catheter. Am J Surg.

[B11] Fagon JY, Novara A, Stephan F, Girou E, Safar M (1994). Mortality attributable to nosocomial infections in the ICU. Infect Control Hosp Epidemiol.

[B12] Doig CJ, Zygun DA, Fick GH, Laupland KB, Boiteau PJ, Shahpori R, Rosenal T, Sandham JD (2004). Study of clinical course of organ dysfunction in intensive care. Crit Care Med.

[B13] Costel EE, Mitchell S, Kaiser AB (1985). Abbreviated surveillance of nosocomial urinary tract infections: a new approach. Infect Control.

[B14] Knaus W, Draper E, Wagner D, Zimmerman J (1985). APACHE II: A severity of disease classification system. Crit Care Med.

[B15] Cullen D, Civetta J, Briggs B, Ferrara L (1974). Therapuetic interventions scoring system: a method of quantitative comparision of patient care. Crit Care Med.

[B16] Semeniuk H, Noonan J, Gill H, Church D (2002). Evaluation of the Coral UTI Screen system for rapid automated screening of significant bacteriuria in a regional centralized laboratory. Diagn Microbiol Infect Dis.

[B17] Martinez OV, Civetta JM, Anderson K, Roger S, Murtha M, Malinin TI (1986). Bacteriuria in the catheterized surgical intensive care patient. Crit Care Med.

[B18] Laupland KB (2004). Population-Based Epidemiology of Intensive Care: Critical Importance of Ascertainment of Residency Status. Critical Care.

[B19] Laupland KB, Church DL, Gregson DB (2003). Evaluation of a rapid bacterial ATP assay for screening BAL samples from ICU patients submitted for quantitative bacterial cultures. Diagn Microbiol Infect Dis.

